# The influence of early thoracic fusion on the pulmonary function of patients with idiopathic scoliosis in the early period of the second growth peak with different Risser signs

**DOI:** 10.1186/s13018-021-02607-y

**Published:** 2021-07-31

**Authors:** Xiaolin Xu, Shengru Wang, Yang Yang, You Du, Guanfeng Lin, Jianguo Zhang

**Affiliations:** 1 Department of Surgery, Peking Union Medical College Hospital (PUMCH), Chinese Academy of Medical Sciences, Beijing, China; 2 Department of Orthopaedic Surgery, Peking Union Medical College Hospital (PUMCH), Chinese Academy of Medical Sciences, No. 1 Shuaifuyuan Hutong, Beijing, 100730 People’s Republic of China

**Keywords:** Early thoracic fusion, Idiopathic scoliosis, Pulmonary function, Second growth peak, Risser sign

## Abstract

**Background:**

Previous reports confirmed early spinal fusion may compromise pulmonary function and thoracic development in skeletal immature patients with scoliosis. However, the different effects in patients with various Risser signs remain unknown. This study aimed to compare the influence of early thoracic fusion on pulmonary function and thoracic growth in patients with idiopathic scoliosis (IS) with closed triangular cartilage (TRC) and different Risser signs.

**Methods:**

Thirty-six patients with IS and a closed TRC were retrospectively selected and divided into the low Risser (LR, Risser sign ≤2, 22 patients) and high Risser (HR, 2<Risser sign≤4, 14 patients) groups. Patient age, Risser sign, main Cobb angle, thoracic kyphosis, and fusion levels were recorded. Perioperative and minimum of 2-year follow-up pulmonary function and thoracic diameters were compared between both groups.

**Results:**

There were no differences in patients’ general characteristics between two groups. The preoperative forced expiratory volume in 1 s (FEV1) and forced vital capacity (FVC) were 2.06±0.43 L and 2.50±0.49 L, respectively, in the LR group, and 2.31±0.49 L (*p* = 0.067) and 2.74±0.56 L (*p* = 0.122), respectively, in the HR group. While these values significantly increased postoperatively, to 2.62±0.46 L (*p* < 0.001) and 3.09±0.69 L (*p* < 0.001), in the LR group, they remained unchanged in the HR group [2.53±0.56 L (*p* = 0.093) and 2.70±0.98 L (*p* = 0.386), respectively]. The FEV1/FVC in both groups was >80% before and after surgery. The T1-T12 and anteroposterior thoracic diameter significantly increased after surgery in both groups, while the maximum inner chest diameter only increased in the LR group at the final follow-up. However, there were no significant differences in respiratory function and thoracic data between both groups.

**Conclusion:**

For patients with IS, early fusion did not deteriorate pulmonary function or thoracic development in TRC-closed patients whose Risser sign was ≤2 compared with those with a Risser sign >2.

## Background

Scoliosis is defined as the lateral curvature of the spine ≥10° in the coronal plane. It is a three-dimensional spinal deformity that usually presents as distortion of the body, thorax, and ribs, thus restricting pulmonary system development [1].

As the most common type of structural scoliosis, the incidence of idiopathic scoliosis (IS) is approximately 1.5% among all teens, while infants with infantile scoliosis represent nearly 4% of patients with IS [2]. Children with progressive curves at a young age are at risk of severe pulmonary issues, attributable to spinal deformity and surgical management [3]. Thus, IS treatment in growing children is challenging since potential harm caused by scoliosis correlates closely with skeletal maturity.

Previous studies found that early spinal fusion may halt progressive deformity in young children with scoliosis, but it does not facilitate lung growth and can result in thoracic insufficiency syndrome in certain children; however, these studies mainly focused on early onset scoliosis (EOS), particularly congenital scoliosis (CS) [4–8], wherein pre-existing factors, including rib malformations, respiratory muscular disorders, or syndrome-related complications in CS and other forms of EOS may play a role in respiratory function. Meanwhile, the skeletal maturity is usually evaluated based on Risser signs and the condition of the triangular cartilage (TRC) [9, 10]. As remaining growth is a determining factor for the worsening of IS, evaluating the growth potential of young patients before therapeutic selection is vital as the higher is the risk of progression, the more severe will be the disease.

To our knowledge, the effects of early thoracic fusion in patients with IS with different skeletal maturities remain unclear, especially after TRC closure, which occurs in the early period of the second growth peak. We hypothesized that patients with lower Risser signs may demonstrate deteriorated pulmonary function as well as thoracic development compared with those with higher Risser signs if they underwent early spinal fusion. This study aimed to analyze and compare the long-term effects of early thoracic fusion on pulmonary function and thoracic growth in patients with IS with a closed TRC and different Risser signs, to provide potential references for interventions in young patients with IS.

## Methods

**Fig. 1:**
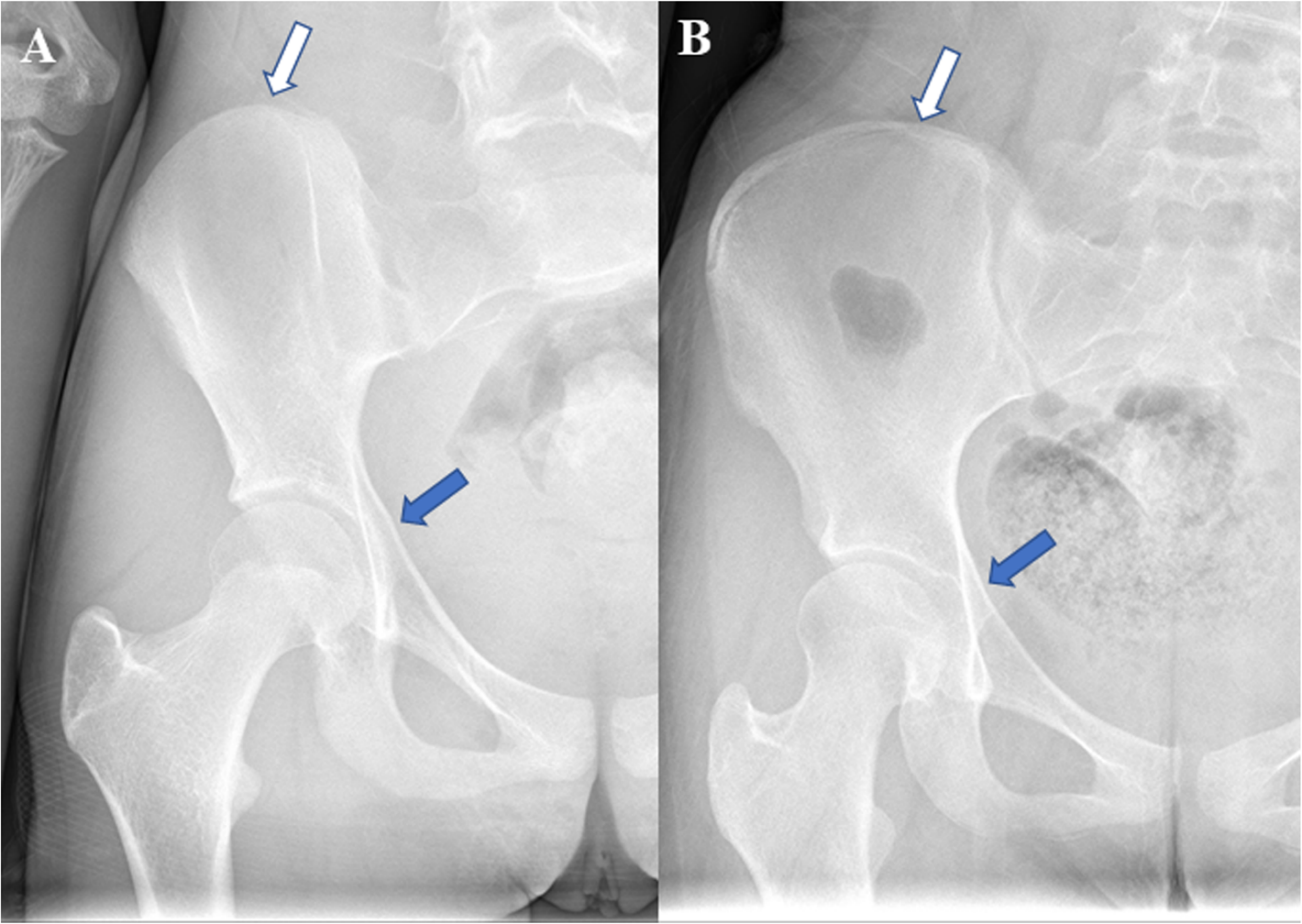
Triangular cartilages and Risser signs. **A** Preoperative image of patients with Risser sign 0 (white arrow) and closed triangular cartilages (blue arrow). **B** Image of patients with Risser sign 5 (white arrow) and closed triangular cartilages (blue arrow) on final follow-up

We retrospectively collected the pre- and postoperative data of patients diagnosed with IS who underwent posterior thoracic fusion between September 2015 and September 2017 in the Department of Spinal Surgery, Peking Union Medical College Hospital (PUMCH). Medical records were reviewed for age at surgery, sex, PUMC classification of scoliosis, extent of thoracic fusion, and maximum follow-up years. Radiographs were also reviewed for preoperative Risser signs, main Cobb angles, and thoracic kyphosis before and after surgery and at follow-up. Patients selected in the study were grouped into the LR group (Risser ≤2, including Risser 0, 1, and 2) and the HR group (2<Risser ≤4, including Risser 3 and 4) based on their preoperative Risser signs (Fig. 1). The Risser sign in our study was based on the definition of the USA six-stage Risser sign grading system in which the iliac crest apophysis is divided into quarters [11]. All patients were between 9 and 12 years old with closed TRC at the time of admission, diagnosed with IS, and underwent one-time posterior fusion surgery related to the thoracic spine. A minimum 2-year postoperative follow-up was required for each patient. All patients reached Risser sign of 5 at the final follow-up. Patients with incomplete medical records, who were unable to complete the follow-up; had primary cardiopulmonary disease such as congenital heart defect and diaphragmatic hernias; or had severe postoperative complications such as pulmonary infection or spinal injury were excluded. Patients with rib abnormalities, such as rib fusions or absent ribs, were also excluded.

Standard pulmonary function tests (PFTs) were recalled, and forced expiratory volume in 1 s (FEV1), forced vital capacity (FVC), and FEV1/FVC were recorded both before surgery and at follow-up. Total lung capacity (TLC) and vital capacity (VC) were recorded at follow-up. All PFTs included the predicted and measured values. The predicted value indicates the normal ventilation parameters of the population, consistent with the sex, age, body weight, and height of the patient. The deterioration of respiratory function included restrictive pulmonary disease, which was defined as FVC less than 50% of predicted value. Meanwhile, the FEV1 and FEV1/FVC values are critically important in the diagnosis of obstructive and restrictive pulmonary diseases as well. Thus, the FEV1/FVC less than 70% and the FEV1 less than 80% of predicted value were also considered impaired respiratory function. All the pulmonary function tests were performed using MasterScreen Pneumo (Erich Jaeger® GmbH & Co KG, Würzburg, Germany), which can provide high measurement precision according to previous report [12].

**Fig. 2:**
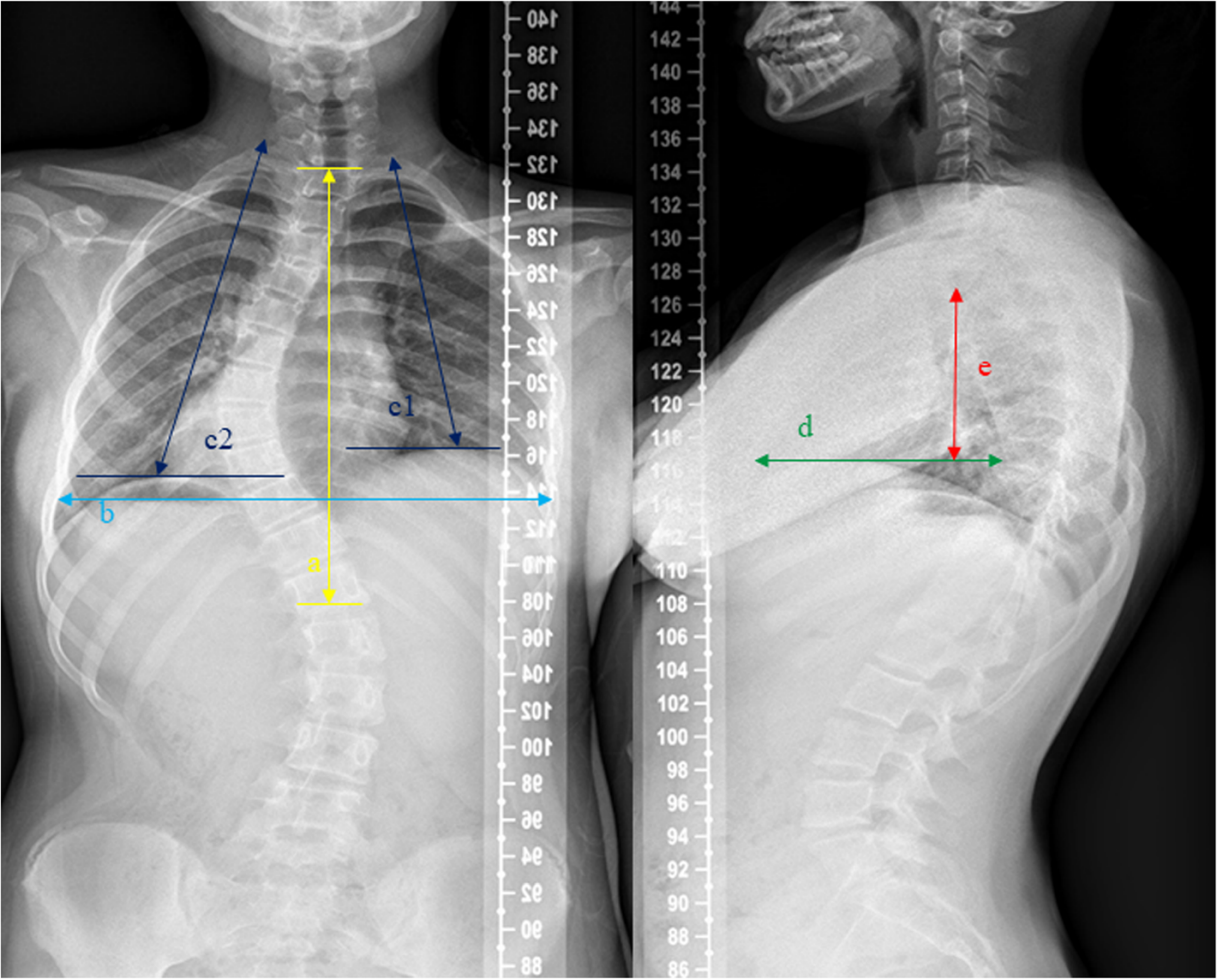
Measurements of the thoracic cage. **a** T1-T12 height. The straight line distance between the midpoint of the upper endplate at T1 and the midpoint of the lower endplate at T12. **b** Maximum inner chest diameter (MICD), the maximum linear distance between the medial margin of the ribs. **c1**, **c2** Campbell’s space available for lung ratio (SAL), the ratio of the distance between the concave side (**c1**) and the convex side (**c2**) of the lateral curvature from the first costal apex to the highest point of the diaphragm. **d** Anterior and posterior thoracic diameter (APTD), the distance between anterior chest wall and the anterior border of the vertebral body through the highest point of the diaphragm on the sagittal plane. **e** Thoracic sagittal longitudinal diameter (TSLD), the distance between the highest point of the diaphragm and the highest point of the chest on the sagittal plane

Spinal height from the 1st thoracic vertebrae (T1) to the 12th thoracic vertebrae (T12) (the straight line distance between the midpoint of the upper endplate at T1 and the midpoint of the lower endplate at T12), maximum inner chest diameter (MICD, the maximum linear distance between the medial margin of the ribs on the coronal plane), Campbell’s space available for lung ratio (SAL, the ratio of the distance between the concave side and the convex side of the lateral curvature from the first costal apex to the highest point of the diaphragm), anterior and posterior thoracic diameter (APTD, the distance between the anterior chest wall and the anterior border of the vertebral body through the highest point of the diaphragm on the sagittal plane), and thoracic sagittal longitudinal diameter (TSLD, the distance between the highest point of the diaphragm and the highest point of the chest on the sagittal plane) were measured (Fig. 2). All the measurement were conducted in Surgimap (Nemaris Inc., Surgimap Ver 2.3.2, New York, NY, USA), which is a reliable and helpful tool that can provide simplified method to evaluate and analyze the spino-pelvic parameters [13]. Two experienced surgeons who were blinded to the outcomes performed all measurements. The mean values were used for the analysis.

Informed consent was obtained from all individual participants included in the study. All procedures performed in studies involving human participants were in accordance with the ethical standards of the institutional research committee (the Peking Union Medical College Hospital Review Board).

### Statistical analysis

The data were analyzed using SPSS (IBM Corp. Released 2013. IBM SPSS Statistics for Windows, version 19.0. Armonk, NY: IBM Corp.) and are expressed as the mean±SD. For the independent samples, the Mann–Whitney *U* test was used to compare data between each group, while the nonparametric test of related samples (Wilcoxon) was used to compare the preoperative, postoperative, and follow-up data in each group. The effect size (ES) of the main variables including the FEV1, FVC, FEV1%, FVC%, FEV1/FVC, T1-T12 height, and MICD of the two groups were calculated. The receiver operating characteristic (ROC) curve for the follow-up data of FEV1%, FVC%, FEV1/FVC, T1-T12 height, MICD, and APTD were also demonstrated; the area under the curve (AUC) of each variable was calculated. The sensitivity and specificity of each variable was also obtained based on the cutoff point. Differences were considered statistically significant at *p* <0.05.

## Results

### General data

**Fig. 3:**
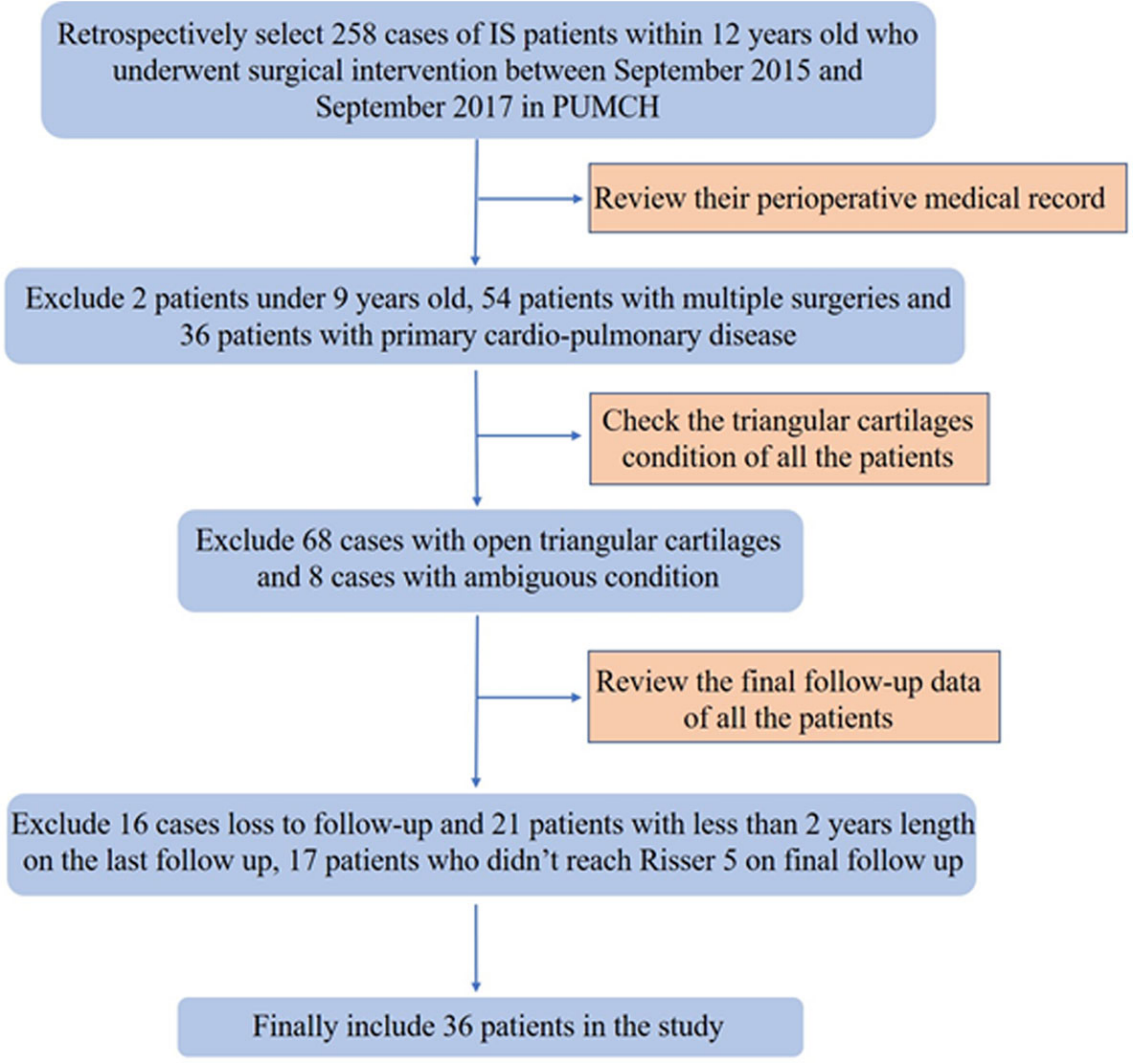
Patient selection. IS, idiopathic scoliosis; PUMCH, Peking Union Medical College Hospital

A total of thirty-six patients reached the inclusive criteria; the selection procedure is illustrated in Fig. 3. Twenty-two patients met the criteria for the LR group, with a mean age of 11.3±0.7 years old and follow-up time of 3.5±1.5 years. The HR group included fourteen patients, with a mean age of 11.8±0.4 years and follow-up time of 3.5±1.4 years. Both groups had similar thoracic fusion proportions. The mean preoperative main Cobb angle was 46.0±10.1°, with a thoracic kyphosis of 21.1±13.6° in the LR group, which were similar to those of the HR group [46.9±7.4° (*p* = 0.275) and 21.5±10.3° (*p* = 0.670), respectively]. On final review, the Cobb angle and thoracic kyphosis decreased to 8.1±6.6° and 17.7±9.7°, respectively, in the LR group, and 9.5±6.6° and 18.2±4.7°, respectively, in the HR group. There was no significant difference between both groups (Table 1).

**Table 1:** General data of two groups

Index	LR group (Risser≤2)	HR group (Risser>2)	***P***
**Cases (number)**	22	14	/
**Age (year)**	11.3±0.7	11.8±0.4	0.083
**Follow-up time (year)**	3.5±1.5	3.5±1.4	0.846
**Thoracic fusion proportion (%)**	80.1±11.7	80.9±10.6	0.961
**Preoperative main cobb angel (°)**	46.0±10.1	46.9±7.4	0.275
**Preoperative thoracic kyphosis (°)**	21.1±13.6	21.5±10.3	0.670
**Follow-up main cobb angel (°)**	8.1±6.6	9.5±6.6	0.380
**Follow-up thoracic kyphosis (°)**	17.7±9.7	18.2±4.7	0.449

*LR* low Risser sign, *HR* high Risser sign

*P* < 0.05 was significant

Patients in the LR group were classified as follows: two PUMC-Ia, one PUMC-IIa, three PUMC-IIb, one PUMC-IIc, one PUMC-IId, and four PUMC-IIIa patients. The HR group included three PUMC-Ia, four PUMC-IIa, five PUMC-IIb, one PUMC-IIc, three PUMC-IId, and six PUMC-IIIa patients.

### Pulmonary function

#### Comparison of predicted and measured values

**Fig. 4:**
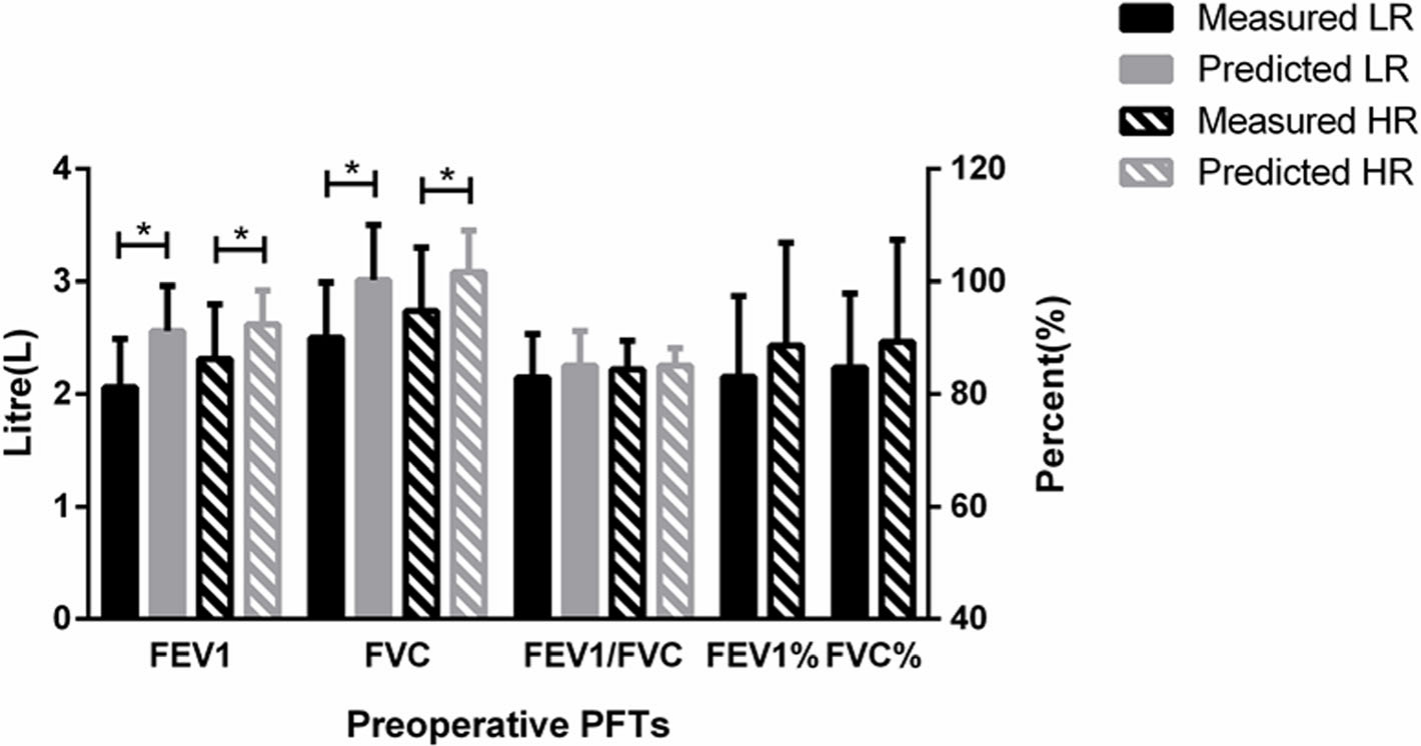
Preoperative pulmonary function tests (PFTs). FEV1, forced expiratory volume in 1 s; FVC, forced vital capacity; FEV1%, the percentage predicted values of FEV1; FVC%, the percentage predicted values of FVC.*p < 0.05

In the LR group, the preoperative FEV1 and FVC were 2.06±0.43 and 2.50±0.49 L, respectively, which was significantly lower than the predicted values [2.56±0.40 (*p* = 0.000) and 3.01±0.49 L (*p* = 0.000), respectively]. In the HR group, the preoperative FEV1 and FVC were 2.31±0.49 and 2.74±0.56 L, respectively, which were lower than the predicted values [2.62±0.30 (*p* = 0.031) and 3.08±0.37 L (*p* = 0.036), respectively] (Fig. 4).

The follow-up results were still significantly lower than the predicted values in both groups. In the LR group, the FEV1 and FVC were 2.62±0.46 and 3.09±0.69 L, respectively, which were significantly lower than the predicted values [3.09±0.45 (*p* = 0.002) and 3.54±0.54 L (*p* = 0.022), respectively], at follow-up. Correspondingly, the FEV1 and FVC were 2.53±0.56 and 2.70±0.98 L, respectively, in the HR group, which were significantly lower than the predicted values [3.07±0.24 (*p* = 0.006) and 3.48±0.31 L (*p* = 0.011), respectively].

**Fig. 5:**
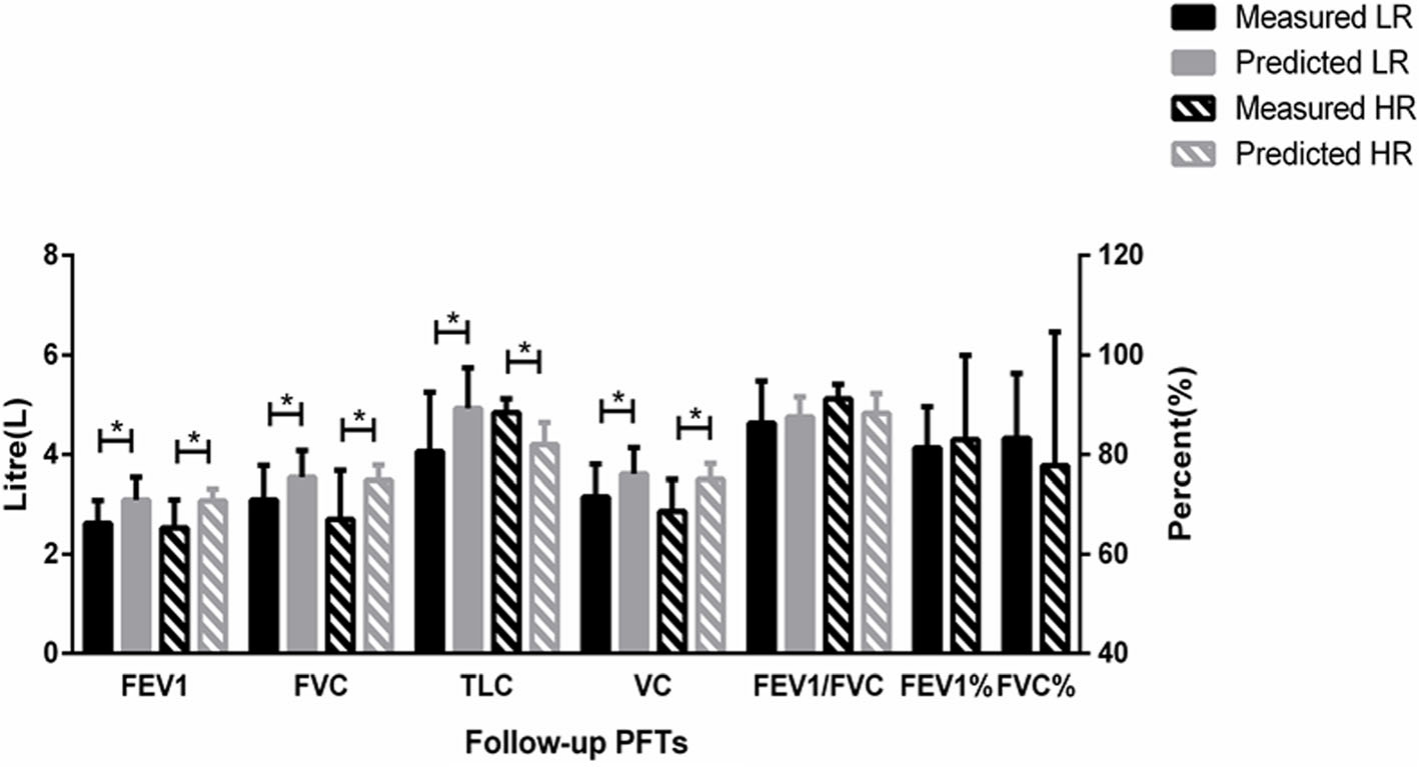
Follow-up pulmonary function tests (PFTs). FEV1, forced expiratory volume in 1 s; FVC, forced vital capacity; TLC, total lung capacity; VC, vital capacity; FEV1%, the percentage predicted values of FEV1; FVC%, the percentage predicted values of FVC. *p < 0.05

The FEV1/FVC showed no significant difference compared with the predicted value in both groups (preoperative: LR 82.93±7.72% vs 85.05±6.12%, *p* = 0.144; HR 84.38±5.07% vs 85.02±3.12%, *p* = 0.609; follow-up: LR 86.28±8.48% vs 87.57±4.00%, *p* = 0.523; HR 91.23±2.88% vs 88.30±3.94%, *p* = 0.644) (Fig. 5).

At follow-up, the predicted TLC and VC were 4.79±0.80 and 3.61±0.53 L, respectively, in the LR group, which were significantly higher than the measured values [3.90±0.99 (*p* = 0.039) and 3.15±0.66 L (*p* = 0.026), respectively]. Similarly, in the HR group, the predicted TLC and VC were 4.70±0.31 and 3.50±0.32 L, respectively, while the measured values were 4.27±0.42 (*p* = 0.024) and 2.86±0.64 L (*p* = 0.004), respectively.

#### Comparison of preoperative and follow-up values

The LR group had significantly increased FEV1 and FVC at the follow-up compared with preoperative results [2.62±0.46 vs 2.06±0.43 L (*p* < 0.00) and 3.09±0.69 vs 2.50±0.49 L (*p* < 0.00), respectively]. However, there was no significant difference between preoperative and follow-up FEV1 and FVC in the HR group.

The percentage predicted values of FVC (FVC%) in both groups were > 50% and demonstrated no significance compared between follow-up and preoperative data [LR 84.70±13.19% vs. 83.28±13.02% (*p* = 0.778); HR 89.28±18.12 vs. 77.74±26.93%, (*p* = 0.214)]. The percentage predicted values of FEV1 (FEV1%) were > 80% in both groups, and no difference was found on final follow-up [LR 83.04±14.39% vs. 81.31±8.29% (*p* = 0.925); HR 88.59±18.24 vs. 82.98±16.94%, (*p* = 0.260)]. Similarly, the FEV1/FVC was also >80% in both groups, regardless of preoperative or follow-up tests. The follow-up results did not differ with the preoperative values in both groups [LR 86.28±8.48% vs. 82.93±7.72% (*p* = 0.147); HR 91.23±2.88 vs. 84.38±5.07%, (*p* = 0.260)].

#### Values compared between groups

None of the PFTs significantly differed between both groups in the preoperative and follow-up tests.

### Thoracic growth and development

#### Comparison between preoperative and postoperative and follow-up diameters

The preoperative T1-T12 height in the LR and HR groups were 235.6±16.3 and 244.7±20.4 mm, respectively. These were significantly increased to 257.2±14.5 (*p* = 0.00) and 263.8±22.6 mm (*p* = 0.00) postoperatively, but remained at 258±14.1 (*p* = 0.00) and 259.1±13.8 mm (*p* = 0.00) at the follow-up (*p=*compared with preoperative value).

The preoperative APTD was 143.7±18.6 and 147.7±20.0 mm in the LR and HR groups, respectively, which increased to 155.3±17.5 (*p* = 0.001) and 155.0±14.5 mm, respectively, (*p* = 0.047) postoperatively. The LR and HR groups also demonstrated a significant increase at follow-up compared with postoperative measurements [162.9±18.6 (*p* = 0.008) and 163.8±15.1 mm (*p* = 0.007), respectively].

The preoperative MICD of the LR and HR groups were 213.0±15.9 and 221.2±16.8 mm, similar to the postoperative values [214.9±16.6 (*p* = 0.393) and 220.5±16.1 mm (*p* = 0.700), respectively]. However, at follow-up, this result significantly increased in the LR group [221.9±15.0 mm (*p* = 0.002)], while that of the HR group remained at 221.7±14.4 mm (*p* = 0.574) compared to preoperative results.

The preoperative SAL was 92.5±3.1% in the LR and 92.7±3.5% in the HR group, while the TSLD was 74.9±14.7 mm in the LR and 77.7±15.6 mm in the HR group. The SAL and TSLD were similar to postoperative and follow-up results in both groups (Table 2).

**Table 2:** Thoracic cage parameters of two groups.

Parameters		Preoperative	Postoperative	Follow up	***P1***	***P2***	***P3***
**LR group (Risser≤2)**	**T1-T12**	235.6±16.3	257.2±14.5	258±14.1	0.000*	0.681	0.000*
	**SAL**	92.5±3.1	94±3.3	95.0±3.7	0.144	0.247	0.727
	**MICD**	213.0±15.9	214.9±16.6	221.9±15.0	0.393	0.002*	0.002*
	**APTD**	143.7±18.6	155.3±17.5	162.9±18.6	0.001*	0.008*	0.000*
	**TSLD**	74.9±14.7	72.5±13.1	73.1±14.1	0.142	0.829	0.441
**HR group (Risser>2)**	**T1-T12**	244.7±20.4	263.8±22.6	259.1±13.8	0.000*	0.201	0.000*
	**SAL**	92.7±3.5	94.7±4.1	96.1±4.2	0.079	0.773	0.104
	**MICD**	221.2±16.8	220.5±16.1	221.7±14.4	0.700	0.107	0.574
	**APTD**	147.7±20.0	155.0±14.5	163.8±15.1	0.047*	0.007*	0.000*
	**TSLD**	77.7±15.6	75.7±14.7	74.2±13.5	0.304	0.370	0.052

*LR* low Risser sign, *HR* high Risser sign, *T1-T12* the straight-line distance between the midpoint of the upper endplate at T1 and the midpoint of the lower endplate at T12, *SAL*, the ratio of the distance between the concave side and the convex side of the lateral curvature from the first costal apex to the highest point of the diaphragm, *MICD* the maximum linear distance between the medial margin of the ribs on the coronal plane, *APTD* the distance between anterior chest wall and the anterior border of the vertebral body through the highest point of the diaphragm on the sagittal plane, *TSLD* the distance between the highest point of the diaphragm and the highest point of the chest on the sagittal plane, *P1* postoperative compared with preoperative values, *P2* postoperative compared with follow-up values, *P3* follow-up compared with preoperative values

*p < 0.05 was considered significant

#### Comparison of diameters between groups

None of the thoracic diameters showed any significant difference between the groups on preoperative, postoperative, or follow-up tests.

### Effect size and ROC curve of main variables

We calculated the effect size of the main variables including the FEV1, FVC, FEV1%, FVC%, FEV1/FVC, T1-T12 height, and MICD of two groups. As the two groups contained different sample size, *Hedges’ g*, which provides a measure of effect size weighted according to the relative size of each sample, was calculated. For preoperative data, the effect size of FEV1 was 0.550808, FEV1% was 0.347439, FVC was 0.463425, FVC% was 0.300357, FEV1/FVC was 0.212321, T1-T12 height was 0.506163, and MICD was 0.504615. For follow-up data, the effect size of FEV1 was 0.179784, FEV1% was 0.135065, FVC was 0.479594, FVC% was 0.283613, FEV1/FVC was 0.717567, T1-T12 height was 0.07865, and MICD was 0.013538.

**Fig. 6:**
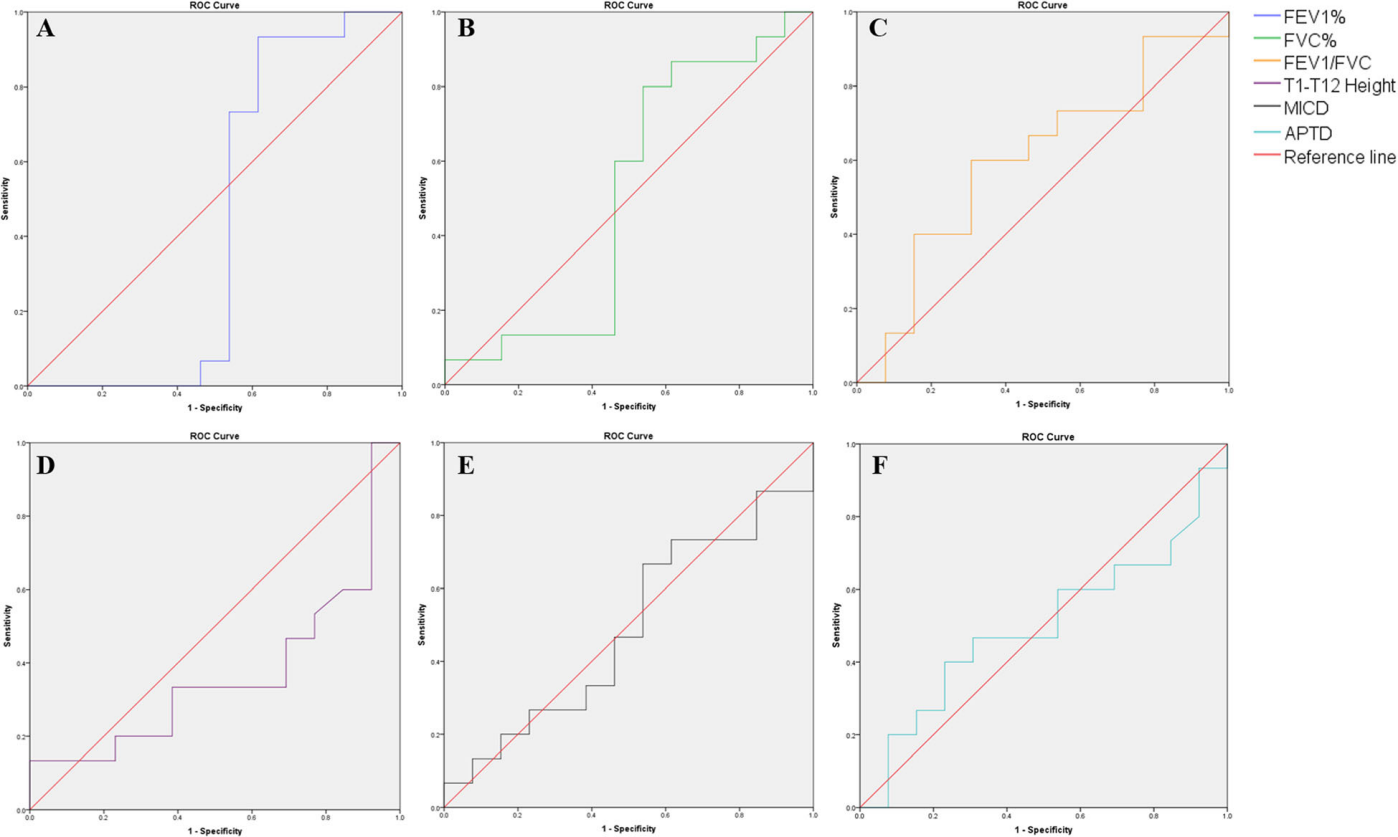
The receiver operating characteristic (ROC) curve of main variables. FEV1%, the percentage predicted values of forced expiratory volume in 1 s; FVC%, the percentage predicted values of forced vital capacity. T1-T12 height, the straight line distance between the midpoint of the upper endplate at T1 and the midpoint of the lower endplate at T12; MICD, the maximum linear distance between the medial margin of the ribs; APTD, anterior and posterior thoracic diameter, the distance between the anterior chest wall and the anterior border of the vertebral body through the highest point of the diaphragm on the sagittal plane. **A** ROC curve of FEV1%. **B** ROC curve of FVC%. **C** ROC curve of FEV1/FVC. **D** ROC curve of T1-T12 height. **E** ROC curve of MICD. **F** ROC curve of APTD

The ROC curve for the follow-up variables including FEV1%, FVC%, FEV1/FVC, T1-T12 height, MICD, and APTD is illustrated in Fig. 6 (from 6A to 6F). The area under the curve (AUC) of each variable was as follows: AUC of FEV1% 0.431 (*p* = 0.534, sensitivity 0.933, specificity 0.385), AUC of FVC% 0.508 (*p* = 0.945, sensitivity 0.800, specificity 0.462), AUC of FEV1/FVC 0.600 (*p* = 0.369, sensitivity 0.600, specificity 0.692), AUC of T1-T12 0.367 (*p* = 0.231, sensitivity 0.133, specificity 1.000), AUC of MICD 0.487 (*p* = 0.908, sensitivity 0.667, specificity 0.462), AUC of APTD 0.500 (*p* = 1.000, sensitivity 0.400, specificity 0.769). The *p* value of all the main variables demonstrated no significance.

## Discussion

This study retrospectively included patients with IS and different Risser signs who underwent thoracic fusion at our hospital after TRC closure. These patients had a similar preoperative age, main Cobb angle, thoracic kyphosis, fusion segments, and follow-up period. Upon comparing the preoperative and follow-up pulmonary function and thoracic growth diameters in the LR and HR groups, we found that (1) all patients demonstrated compromised preoperative FEV1 and FVC compared to the predicted values. (2) After early thoracic fusion, although the FEV1 and FVC were still lower than the predicted values in both groups, they increased significantly in the LR group compared with the preoperative results. (3) The FEV1/FVC in both groups was >80% regardless of preoperative or follow-up tests, which did not change after spinal fusion. (4) The T1-T12 height and APTD in both groups increased significantly after thoracic fusion. However, compared with the postoperative results, the follow-up T1-T12 height showed no increase, while the APTD increased significantly. (5) In the LR group, the MICD also increased at the final follow-up. (6) All PFTs and thoracic parameters were similar between both groups on preoperative, postoperative, and follow-up measurements.

The clinical strategies for IS in very young children are extremely challenging. For mild to moderate scoliosis, conservative treatments such as physiotherapy, casting, and bracing are mostly recommended for juvenile patients, which can slow down the progression of the deformity until spine fusion can be performed [14, 15]. However, spinal deformity in this group is often relentlessly progressive; thus, in addition to casting and bracing, spinal fusion with instrumentation is usually indicated for patients with progressive scoliosis >50° [16–18], as well as in primary thoracic curves >30° at the beginning of puberty if patients show progress during the first year of pubertal growth [19]. This treatment is undertaken with the belief that a spine that is “short and straight” would be better in the long term than a spine with further curve progression that is “longer but crooked” [20, 21]. Newer concepts affirm the importance of maintaining spine growth and minimizing curvature simultaneously; thus, “growth-friendly” systems are promoted [22–24].

The influence of early spinal fusion in young patients possibly involves three aspects: spinal deformity, thoracic growth, and pulmonary function. Previous studies have shown that deformity progression in CS may not be well-controlled by early fusion because of the high revision surgery rate, pseudarthroses, and adding-on phenomenon at the cephalad or caudal ends of the initial fusion [4, 6, 25]. However, in our study, both groups demonstrated similar preoperative Cobb angles and kyphosis after undergoing similar extension of thoracic fusion, and the follow-up assessment demonstrated similar significantly reduced main Cobb angle and kyphosis, indicating that both groups had well-controlled deformities.

In thoracic growth, IS itself can have negative effects on the growing spine as asymmetrical forces act on the growth plates of the vertebral column [15]. When managing severe and very severe scoliosis in young patients, posterior instrumented fusion is a rational choice because correction of the deformity compensates for the overall loss of spinal height [15]. In 2008, Bowen et al. found that patients with CS who had fusions before the age of 5 years demonstrated a 0.48-cm increase per year in the T1-T12 height, which was nearly half of those who did not [5]. For normal growth, the length from T1-T12 averages at 18 cm at 5 years of age, 22 cm at 10 years of age, and 26.5–28 cm in adults, based on the studies by Dimeglio et al. [26, 27]. This is consistent with our results, in which the preoperative T1-T12 was approximately 23.5±1.6 and 24.4±1.6 cm in the LR and HR groups, respectively. At the final follow-up, although lower than adult levels were reported by Dimeglio et al., these levels increased significantly to 25.8±1.4 and 25.9±1.4 cm, and the annual growth rate of T1-T12 was 0.64 cm and 0.41 cm for the LR and HR groups, respectively. In contrast, in both the LR and HR groups, the T1-T12 height and APTD increased significantly after thoracic fusion. Compared with the postoperative results, the follow-up T1-T12 height in both groups showed no increase, while APTD still increased significantly. This indicated that after TRC closure, patients who had thoracic fusion may remain at a steady T1-T12 height, but the anteroposterior diameter of the thoracic cage may continue to increase. More importantly, compared with the preoperative value, postoperative MICD in the LR group remained unchanged, but the follow-up MICD increased significantly. This revealed that patients with low Risser signs may maintain their growth potential in the thoracic width, while those with high Risser signs may not.

A progressive spinal deformity leads to thoracic cage distortion, and more importantly, compromise lung growth [15]. Puberty is a turning point in children with IS as the pubertal growth spurt increases the risk of progression. Since Risser 0 makes up 2/3 of puberty, the Risser 0 period must be divided into two parts: Risser 0-Triradiate Cartilage open and Risser 0-Triradiate Cartilage closed [15]. The reason we limited the scope to closed TRCs was to exclude patients with extremely low age. In addition, for a healthy child, the greatest increase in the number of alveoli occurs in the first 2 years of life [28, 29], and the multiplication of alveoli is typically completed by the age of 8 years [6]. Further, there are significant barriers to the acquisition of PFTs in young patients, including a short attention span and difficulty in cooperating [30, 31]. Thus, we included patients over 9 years of age to avoid the influence of different bases of lung growth.

Previous studies have demonstrated the frustrating effects of early spinal fusion on pulmonary function, mainly in patients with EOS. Goldberg et al. reported that in 11 patients fused before 10 years of age, the FEV1 and FVC both averaged to 41% of normal values on the final follow-up when patients reached at least 15 years of age, while these increased to 79% and 68%, respectively, in those who were fused at an older age. Furthermore, those whose scoliosis resolved or were stabilized by nonoperative means demonstrated normal levels of FEV1 and FVC (98.7% and 96.6% of normal) [7]. In another study by Karol et al., 28 patients with a mean age of 3.3 years underwent spinal fusion, after an average 11-year follow-up. They found that the average FVC was 57.8% and FEV1 was 54.7% of normal values. Further, in 12 of the 28 patients, the FVC was <50% of the normal value. They concluded that fusion of the proximal aspect of the spine (T1 or T2) and more than four segments were associated with diminished pulmonary function [4]. Vitale et al. compared the difference between early thoracic fusion and thoracolumbar fusion in infantile patients with congenital scoliosis. They found that the predicted FVC averaged at 64.2% and 88.5% for patients with thoracic and lumbar fusions, respectively, while the thoracic fusion group also showed lower scores on quality of life [8].

These studies included patients with various Cobb angles and fusion segments, and the types of scoliosis as well as the associated abnormalities were also inconsistent among groups, especially in patients with CS. In our study, only patients with IS were included because either congenital or syndrome-related scoliosis may be accompanied by rib malformation or muscular diseases. Previous histochemical and physiological study of IS has also confirmed no evidence of myopathic changes in the paravertebral muscles [32], indicating choosing IS patients as study objective could reduce the influence of muscle-related effects. More importantly, the similarity of patients’ basic information also confirmed the comparability between the two groups in our study. All the patients entered the second growth peak as they demonstrated a closed TRC, in which Risser signs ≤2 occurred in the early stage of this period, the junction of accelerating and decelerating period [15, 19]. Traditionally used to estimate the future growth potential of the spine, the Risser sign grading system is based on the fact that vertebral growth is completed at approximately the same time as fusion of the completely ossified iliac crest apophysis to the iliac bone occurs [11, 15]. Clinically, for a patient with severe scoliosis, if the Risser sign is <2, both fusion and non-fusion surgical intervention may be considered as clinical strategies, and to our knowledge, no research has compared the postoperative pulmonary function in patients who underwent fusion surgery with different Risser signs.

In our study, the average preoperative FEV1 of the LR group was 80.5% and the FVC was 83.1% of normal values; they increased significantly to 84.8% (*p* < 0.000) and 87.3% (*p* < 0.000) at the final follow-up. In the HR group, the preoperative FEV1 was 88.2% and FVC was 89.0% of normal values; however, they showed no significance compared with values [82.4% (*p* =0.093) and 77.6% (*p* =0.386), respectively] observed at final follow-up. Further, the measured TLC and VC were also lower than the predicted values at the follow-up tests. However, although the measured values of FEV1 and FVC were both remarkably lower than the predicted values in the two groups on preoperative and follow-up tests, they all reached over 80% of the predicted value. Most importantly, the significant increase in postoperative FEV1 and FVC in the LR group demonstrated that early thoracic fusion in patients in the early period of the second growth peak with low Risser signs may have improved pulmonary function. This may also be explained by the increase in both the T1-T12 height and APTD through thoracic fusion. In addition, both groups showed normal FEV1/FVC in the preoperative and follow-up results, indicating that restrictive lung disease may not occur in patients with IS who underwent thoracic fusion after the closure of TRC.

This study has several limitations. First, a group of non-surgery or non-fusion patients with similar basic information as the LR and HR groups would make the study better, which requires a prospective study design in the future. Second, the number of patients available for the study was limited, not only due to the inclusion criteria, but also because of patient’s compliance with the postoperative follow-up. Third, we lacked postoperative evaluation results of pulmonary function in this study; further, evaluation of the life quality score and cardiovascular system such as ejection fraction and pulmonary artery pressure should also be assessed in future research design.

## Conclusions

This study demonstrated that early thoracic fusion would not deteriorate thoracic cage development and respiratory function in patients with TRC closed idiopathic scoliosis with low Risser signs (0–2) compared with high Risser signs (3–4) during long-term observation. On the contrary, compared with patients with high Risser signs, early thoracic fusion may improve pulmonary function, especially FEV1 and FVC, in patients whose Risser sign is ≤2, and patients with lower Risser signs may also have a higher growth potential in thoracic development.

## Data Availability

Not applicable.

## Abbreviations

IS: Idiopathic scoliosis
TRC: Triangular cartilages
LR: Low Risser
HR: High Risser
EOS: Early onset scoliosis
CS: Congenital scoliosis
PUMCH: Peking Union Medical College Hospital
PFTs: Pulmonary function tests
FEV1: Forced expiratory volume in 1 s
FVC: Forced vital capacity
TLC: Total lung capacity
VC: Vital capacity
T1: The 1st thoracic vertebrae
T12: The 12th thoracic vertebrae
MICD: Maximum inner chest diameter
SAL: Campbell’s space available for lung ratio
APTD: Anterior and posterior thoracic diameter
TSLD: Thoracic sagittal longitudinal diameter
